# RNA virus spillover from managed honeybees (*Apis mellifera*) to wild bumblebees (*Bombus* spp.)

**DOI:** 10.1371/journal.pone.0217822

**Published:** 2019-06-26

**Authors:** Samantha A. Alger, P. Alexander Burnham, Humberto F. Boncristiani, Alison K. Brody

**Affiliations:** 1 Biology Department, University of Vermont, Marsh Life Sciences, Burlington, VT, United States of America; 2 Honeybee Research and Extension Laboratory, Entomology and Nematology Department, University of Florida, Gainesville, FL, United States of America; University of North Carolina at Greensboro, UNITED STATES

## Abstract

The decline of many bumblebee species (*Bombus* spp.) has been linked to an increased prevalence of pathogens caused by spillover from managed bees. Although poorly understood, RNA viruses are suspected of moving from managed honeybees (*Apis mellifera*) into wild bumblebees through shared floral resources. We examined if RNA viruses spillover from managed honeybees, the extent to which viruses are replicating within bumblebees, and the role of flowers in transmission. Prevalence and active infections of deformed wing virus (DWV) were higher in bumblebees collected near apiaries and when neighboring honeybees had high infection levels. We found no DWV in bumblebees where honeybee foragers and honeybee apiaries were absent. The prevalence of black queen cell virus (BQCV) was also higher in bumblebees collected near apiaries. Furthermore, we detected viruses on 19% of flowers, all of which were collected within apiaries. Our results corroborate the hypothesis that viruses are spilling over from managed honeybees to wild bumblebees and that flowers may be an important route for transmission.

## Introduction

Many infectious diseases are caused by generalist pathogens that infect multiple host species [1]. For pathogens capable of infecting multiple hosts, spillover occurs when the pathogen is introduced and transmitted from a reservoir population into a naive host population. Pathogen spillover between managed and wild animals causes species declines, threatens global biodiversity, and alters ecosystem function and services [2,3].

Given recent declines in managed honeybees (*Apis mellifera*), the importance of native pollinators has risen to global attention [4,5]. Many of the threats to managed honeybees are also affecting native bees [6–9], most notably the increased prevalence of pathogens putatively caused by spillover events from managed bees [10–16]. Although poorly understood, RNA viruses are suspected of moving from managed honeybees to other insects, including wild bees [17,18]. Once considered to be specific to European honeybees, RNA viruses have now been detected in a wide range of insects including bumblebees, solitary bees, hoverflies, wasps and ants [13,17–25]. Due to their high mutation rates and short generation time, RNA viruses are likely to cross species barriers and adapt rapidly to new host environments [18,26]. Both relatedness and shared foraging habits may increase the risk of disease transfer among managed honeybees and wild bumblebees [23,27]. In the United Kingdom (UK), sympatric bumblebees and honeybees are infected by the same deformed wing virus (DWV) strains [17] and virus prevalence in honeybees is a significant predictor of virus prevalence in bumblebees [13]. Although there is some evidence of virus spillover from managed honeybees into wild bees, more work is needed to elucidate transmission routes, the principal directionality of virus transmission, and whether, once contacted, viruses replicate in bumblebee hosts [28].

Horizontal transmission routes for viruses among bee species are currently suspected but largely unconfirmed. Transmission may occur through the use of shared floral resources [13,17,18,29], either by contact with the feces and glandular secretions of worker honeybees or in pollen loads [18,30,31]. However, to our knowledge, only one study examined bee viruses in pollen collected from flowers and this done in a single honeybee apiary [30]. Thus, the degree to which viruses can be horizontally transmitted with flowers acting as a bridge remains poorly understood [13].

Here, we assessed evidence for the spillover of RNA viruses from managed honeybees into wild bumblebees and if transmission is facilitated by the shared use of floral resources. First, we examined the presence (“prevalence” hereafter) of RNA viruses and whether the viruses were actively replicating in two bumblebee species. We then examined if virus prevalence, load, and active infection in bumblebees correlated with the presence of managed honeybee colonies and their virus loads. We also investigated transmission through shared floral resources by testing flowers collected from our field sites and examining how bee abundance, honeybee colony presence, and landscape level floral density influenced patterns of virus prevalence.

## Materials and methods

### Field sampling

To determine the prevalence of RNA viruses in bumblebees and to assess if there is evidence for virus spillover from managed honeybees, we collected bumblebees, honeybees, and flowering plants across Vermont, USA. We chose seven sites with commercial honeybee apiaries within 300 m (S1 Table). We chose twelve sites with no commercial apiaries within 1 km. Published data on bee flight ranges for honey bees and bumble bees indicates a 1 km distance interval provides the most overlap in foraging for sympatric honey bees and bumble bees [32–35]. Sampling was conducted June 18^th^- August 26^th^ 2015. Within a given week of sampling, we went to both apiary and non-apiary sites to ensure that site type was not confounded by time. At each site, we collected up to 15 bumblebees of each target species: *Bombus vagans* and *B*. *bimaculatus* (S1 Table). To reduce collecting multiple samples from the same colony, collections were made throughout the entire sampling area of at least 15,000 m^2^. Honeybees were found in sites with and without apiaries nearby. In sites with apiaries, we sampled bees from eight randomly chosen colonies by netting forager honeybees directly from hive entrances. In sites without a commercial apiary within 1 km, we collected up to 10 honeybee foragers from flowers. Honeybees were entirely absent during sampling in four sites (S1 Table). We placed all bees on dry ice in the field to preserve RNA until lab storage at -80°C. Bee abundance could influence the likelihood of bee-to-bee contact; therefore, we measured honeybee and bumblebee abundance by recording all individuals within 5 m of either side of a 100 m transect over a 10-minute period at each site. Bee abundance surveys were conducted between the hours of 10 a.m. and 5 p.m. Landowners granted permission to conduct the study on the sites and the field study did not involve endangered or protected species.

Because shared flowers are suspected bridges for viruses transmission between bees [18,36], we tested if flowers can harbor viruses by collecting 20–60 inflorescences from the most highly bee-visited and locally common flower species at each site. Entire inflorescences were collected, placed on dry ice in the field, and stored in the lab at -80°C until tested for viruses. At each site, we identified and counted all flowering plants within a 1m x 1m quadrat placed every 10 m along the 100 m bee survey transect [37]. For each site, average flowering plant density was calculated as inflorescences/m^2^.

### Virus detection and quantification

We extracted total RNA following Qiagen RNeasy mini kit protocols. We homogenized individual bumblebees and pooled honeybees (up to 10 per site) in GITC buffer (S1 Appendix) over liquid nitrogen. For plants, we homogenized 1.5 g of flower material in GITC buffer over liquid nitrogen. Qiagen protocols were used to extract RNA. We assessed all RNA quantity and quality using a Spectrometer (Nanodrop, Thermo Scientific).

For reverse transcription of RNA and absolute quantification of each virus in bees and plants, we performed duplicate reverse transcription quantitative polymerase chain reactions (RT-qPCR) for each diluted sample using SYBR green one-step RT-qPCR kit in 10 ul reactions. We used the following thermal cycling program: 10 min at 50°C (RT) followed by 1 min at 95°C, and 40 amplification cycles of 95°C for 15 s, 60°C for 60s and derived melt-curves using the following program: 65–95°C (0.5°C increments, each 2s). We used primers specific to the following RNA virus targets: deformed wing virus (DWV), black queen cell virus (BQCV) and Israeli acute paralysis virus (IAPV), and a housekeeping gene (*Actin*) as a positive control of RNA extraction efficiency in bees (S2 Table). To quantify virus load, we used triplicate standard curves of gBlocks Gene Fragments (Integrated DNA Technologies) (S1 Data). Efficiencies were 91% (DWV), 95% (BQCV), 90% (IAPV), and 90% (*Actin*), with correlation coefficients (R^2^) ranging from 0.993–0.999. We tested a total of 15 composite honeybee samples and 342 bumblebee workers consisting of 180 *B*. *vagans* and 162 *B*. *bimaculatus*. We tested 33 composite flower samples of which 13 were collected from sites with apiaries and 20 were collected from sites without apiaries.

### Negative strand detection

Although bees may pick up viruses on flowers and test positive for the presence of a virus, these are not necessarily active infections. To test for actively replicating viruses in the bumblebees, we conducted strand specific RT-PCR [38] on extracted RNA samples that tested positive for a virus. Each RNA sample was transcribed to cDNA using iScript cDNA Synthesis Kit (BioRad). To increase specificity, we used PAGE purified, biotinylated forward and reverse primers (Integrated DNA Technologies) during reverse transcription and purified the resulting cDNAs using magnetic beads coated with a monolayer of streptavidin following manufacturers protocols (New England BioLabs). We diluted each cDNA tenfold and then conducted PCRs with non-biotinylated primers in separate reactions for both for forward and reverse strands.

### Sequencing

To confirm the identity of the viruses, we sequenced virus fragments from bumblebees and honeybees. qPCR product was cleaned (ExoSAP-IT PCR Product Cleanup) and sequencing was performed using the 3130xl Genetic Analyzer in the University of Vermont Cancer Center Advanced Genome Technologies Core. Sequence data were viewed (FinchTV 1.4) and aligned by eye to genome references using Geneious v 6.0.6 (BQCV: GenBank: KY243932.1; DWV: GenBank: KJ437447.1).

### Data reporting

We use “prevalence” to refer to the percentage of bumblebees positive for a virus. Virus load was measured as average virus genome copies/bee. Virus load results for flowers were measured as virus genome copies/gram of flower material. We binned sites as either ‘high’ (>10^7^) or ‘low’ (<10^7^) honeybee virus loads based on the clear bimodal distribution of the logarithmic value of the virus genome copies/bee at for each site (S1 Fig). To measure honeybee and bumblebee abundance at each site, we calculated the number of bees observed within 5 m of either side of a 100 m transect over a 10-minute period. We calculated floral density as the number of flowering inflorescences per m^2^.

### Data processing and analysis

We analyzed data from the qPCR runs using Thermo Fisher Cloud Software, v 1.0 (Life Technologies Corporation), and R v 0.99.903 (R Core Team 2016). We selected six ten-fold dilutions for each target (DWV, BQCV, IAPV, and Actin) and used a regression analysis to derive a standard curve for quantification.

### Statistics

We performed all analyses in R v 0.99.903 (R Core Team 2016). To analyze bumblebee virus load data, we first log transformed all virus loads to improve normality. To investigate whether honeybee apiary presence, floral density, or bumblebee species affected the prevalence, load, or replication of RNA viruses in bumblebees (DWV and BQCV were tested in separate models), we used separate general linear mixed models (GLMMs) (R library lme4, v 1.1.13, functions lmer and glme) with virus prevalence, virus load, and presence of the viral negative strand as our response variables. To test whether honeybee abundance influenced the prevalence, load, or replication of RNA viruses in bumblebees, we used the same model structure, conducting separate GLMMs and substituting honeybee abundance for honeybee apiary presence. Virus load in bumblebees was analyzed using a Gaussian distribution and the presence of virus as a binomial distribution. In each model we used the fixed effects of apiary absent/present, site level floral density, and bumblebee species with site, latitude and longitude as random effects.

Site average honeybee virus loads were bimodally distributed (S1 Fig). Therefore, we used a separate GLMM with binomial distribution to test if DWV virus prevalence in bumblebees is affected by the virus load in honeybees (high: >10^7^ genome copies; low: < 10^7^ genome copies) or honeybee abundance. We used honeybee viral load, honeybee abundance, and floral density as fixed effects and site as a random effect.

To investigate whether honeybee or bumblebee abundance affects virus deposition on plants, we used a GLMM with binomial distribution with honeybee abundance, bumblebee abundance and virus (DWV, BQCV) as fixed effects and site as a random effect. To examine if the presence of apiaries and bumblebee species affected the prevalence of replicating viruses, separate chi-square tests for independence were conducted for each virus. To calculate the significance of each fixed effect for all models, we created a reduced model by removing the effect, and compared each reduced model to our full model using a likelihood ratio test. All full models tested against a random null model to ensure that full models explained more variance than a random model.

## Results

We detected BQCV in 75.7% and DWV in 9.3% of bumblebees tested (Table 1). We did not detect Israeli acute paralysis in any of the bees. The virus prevalence in honeybees was 100% for both BQCV and DWV, and loads ranged from 10^6^ to 10^9^ for BQCV and 10^4^ to 10^10^ for DWV. Honeybee DWV loads followed a bimodal distribution (S1 Fig) with clear separation between two groups which we designated as either having “low” (< 10^7^ genome copies) or “high” (> 10^7^ genome copies) virus loads. The prevalence of BQCV was significantly higher in *B*. *bimaculatus* (86.3%) compared to *B*. *vagans* (65.9%) (*χ*_1_^2^ = 15.671,P<0.001) but DWV prevalence was similar in both bumble bee species (*χ*_1_^2^ = 0.263,P = 0.608; Table 2).

**Table 1:** Results of virus assays for bumblebees, honeybees, and flowers. Virus loads are presented as the observed range of viral genome copies. Prevalence % is the percentage of samples positive for a virus. Prevalence % (-) is the percentage of bees in which we detected the negative virus strand, indicative of a replicating infection. Site type ‘No *Apis’* are sites without apiaries and with no honeybees observed during sampling.

	Virus load		Prevalence %		Prevalence % (-)	
	BQCV	DWV	BQCV	DWV	BQCV	DWV
***Bombus***	10^4^−10^8^	10^4^−10^7^	75.7	9.3	17.2	6.3
Species:						
*B*. *bimaculatus*	10^4^−10^8^	10^4^−10^5^	86.3	9.3	26.2	7.4
*B*. *vagans*	10^4^−10^8^	10^4^−10^7^	65.9	9.2	8.6	5.2
Site type:						
Apiary present	10^4^−10^8^	10^4^−10^7^	90.5	16.4	20.3	10.3
Apiary absent	10^4^−10^8^	10^4^−10^6^	67.9	5.5	15.5	4.1
No *Apis*	10^4^−10^8^	0	37.5	0	12.1	0
***Apis mellifera***	10^6^−10^9^	10^4^−10^10^	100	100	-	-
**Flowers**	10^3^−10^5^	10^2^−10^6^	15.4	27.3	-	-
Site type:	-	-	-	-	-	-
Apiary present	10^3^−10^5^	10^2^−10^6^	20.0	26.7	-	-
Apiary absent	0	0	0	0	-	-

**Table 2:** Results of the GLMMs showing each model and the fixed effects tested. Table shows chi squared value, degrees of freedom (Df) and p-value. Apiary presence refers to whether the site had a commercial apiary present or no apiary nearby. Floral density was calculated as the number of inflorescences per m^2^. Bee species was either *Bombus bimaculatus* or *B*. *vagans*. Asterisks (*) represent significance.

Model/Parameter	*χ*^2^	Df	P
**BQCV Prevalence**	-	-	-
Apiary Presence	3.959	1	0.047*
Floral Density	0.273	1	0.601
*Bombus* Species	15.67115	1	<0.001*
**DWV Prevalence**	-	-	-
Apiary Presence	6.531	1	0.012*
Floral Density	6.025	1	0.014*
*Bombus* Species	0.263	1	0.608
**BQCV Load**	-	-	-
Apiary Presence	0.943	1	0.331
Floral Density	2.902	1	0.088
*Bombus* Species	18.662	1	<0.001*
**DWV Load**	-	-	-
Apiary Presence	1.064	1	0.302
Floral Density	0.263	1	0.608
*Bombus* Species	0.089	1	0.765
**BQCV Negative Strand**	-	-	-
Apiary Presence	0.134	1	0.715
Floral Density	0.201	1	0.654
*Bombus* Species	15.618	1	<0.001*
**DWV Negative Strand**	-	-	-
Apiary Presence	4.861	1	0.027*
Floral Density	5.461	1	0.019*
*Bombus* Species	0.068	1	0.794

**Fig 1:**
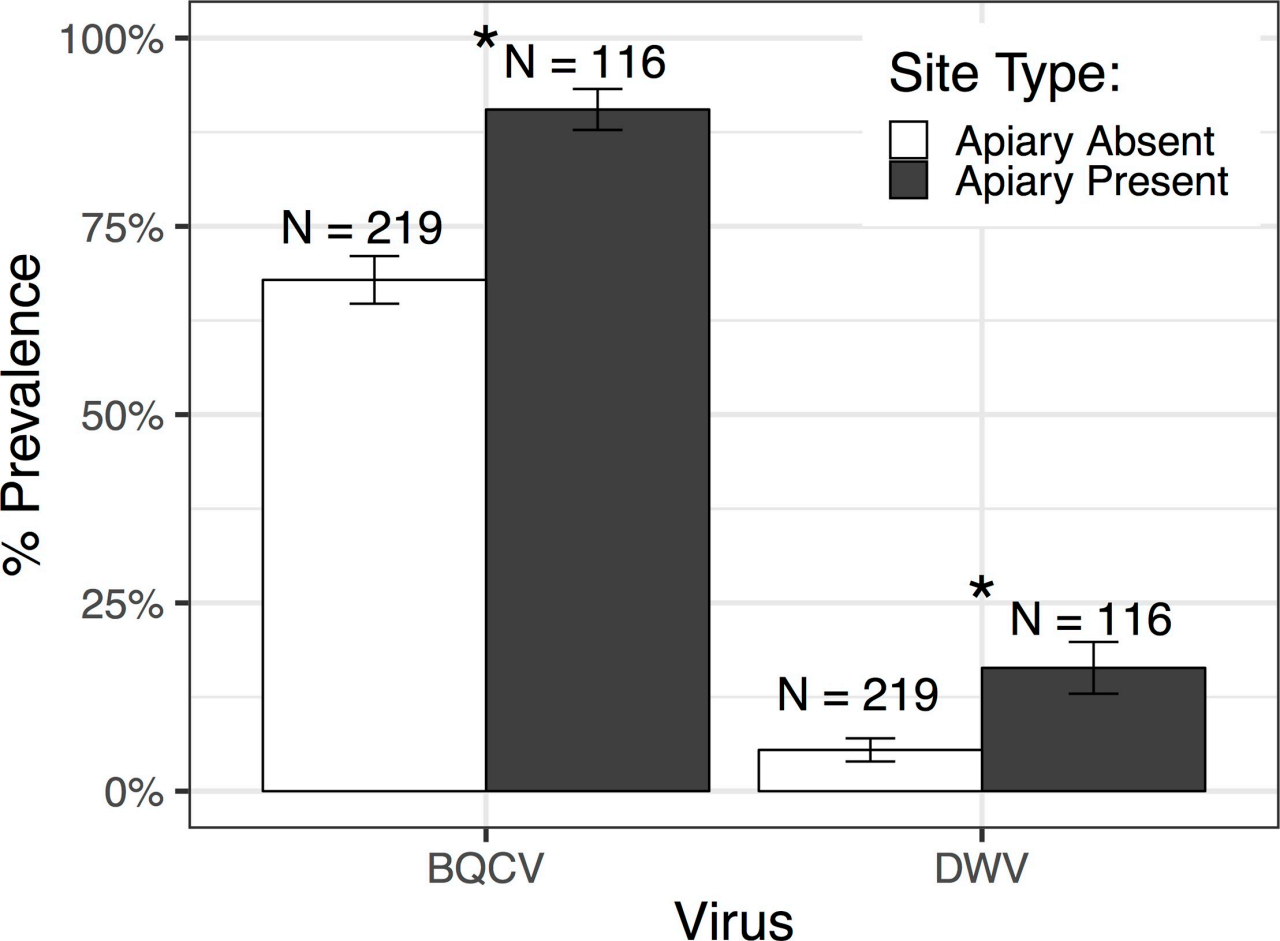
Percent prevalence of infected bumblebee individuals for black queen cell virus (BQCV) and deformed wing virus (DWV). Bumblebees were either caught in sites with honeybee apiaries present or no apiary nearby. BQCV and DWV were more prevalent in bumblebees caught in sites with a honeybee apiary present than in sites without an apiary nearby. Standard error bars are shown. Asterisks represent significance.

**Fig 2:**
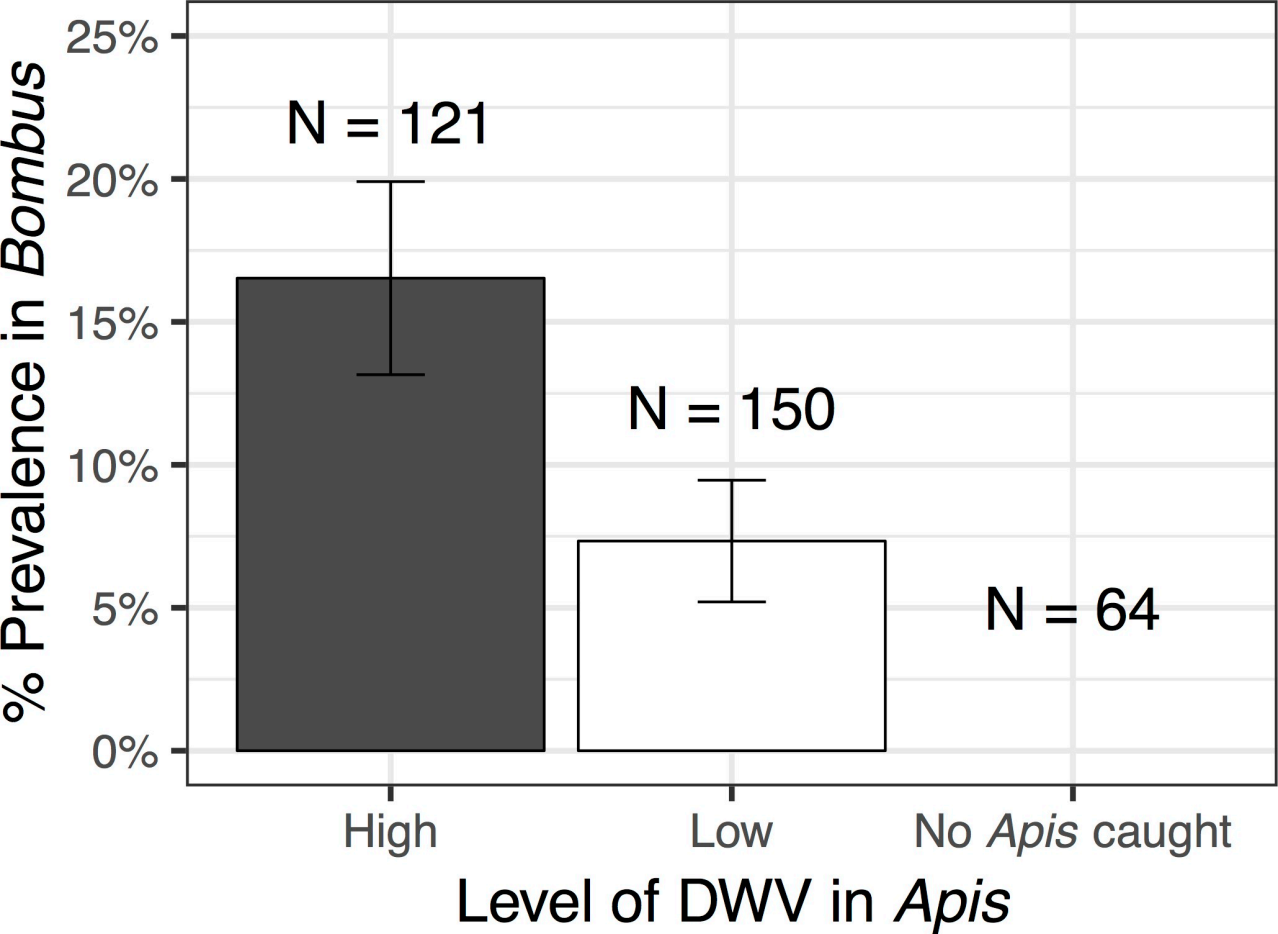
Percent prevalence for bumblebees infected with deformed wing virus (DWV) at sites where honeybees had high and low viral loads, and sites where no honeybees were present and therefore could not be collected. DWV was more prevalent in bumblebees caught at sites with honeybees with high average viral loads, than sites with honeybees with low average viral loads. Standard error bars are shown.

Bumblebees collected within 1 km of a honeybee apiary had significantly higher prevalence of BQCV and DWV compared to bumblebees collected from sites without an apiary nearby (BQCV: *χ*_1_^2^ = 3.959,P = 0.047; DWV: *χ*_1_^2^ = 6.531,P<0.012) (Fig 1). Bumblebee virus prevalence was highest in sites with high honey bee abundance (BQCV: *χ*_1_^2^ = 3.868,P = 0.049; DWV: *χ*_1_^2^ = 5.856,P< 0.016) (S3 Table). In sites without a commercial apiary and with no honeybee foragers observed during collection, 37.5% of bumblebees were positive for BQCV and none were positive for DWV (Fig 2). Virus load was not significantly affected by apiary presence for either virus.

The prevalence of DWV in bumblebees was significantly higher in sites with high honeybee DWV loads compared to bumblebees collected from sites where DWV load in honeybees was low (*χ*_1_^2^ = 8.068,P = 0.018) (Fig 2, S4 Table). Virus loads (virus genome copies per bee) in bumblebees ranged from 10^4^ to 10^7^ for DWV and 10^4^ to 10^8^ for BQCV. *Bombus bimaculatus* had significantly higher virus loads than *B*. *vagans* for BQCV (*χ*_1_^2^ = 18.662,P<0.001) but not DWV (*χ*_1_^2^ = 0.0089,P = 0.765).

By amplifying the negative strand, we detected actively replicating BQCV and DWV in both bumblebee species. We found BQCV replication in 17.2% and DWV replication in 6.3% of bumblebees. Actively replicating BQCV infections were higher in *B*. *bimaculatus* (26.2%) compared to *B*. *vagans* (8.6%) (*χ*_1_^2^ = 15.618,P<0.001). DWV was actively replicating in 7.4% of *B*. *bimaculatus* and 5.2% of *B*. *vagans* (*χ*_1_^2^ = 0.068,P = 0.794). Replicating DWV was more prevalent in bumblebees collected near honeybee apiaries (*χ*_1_^2^ = 4.861,P = 0.027). However, this was not the case for BQCV infections (*χ*_1_^2^ = 0.134,P = 0.715) (Table 2).

Overall, we detected viruses on 19.4% of the flower samples. The virus loads on flowers ranged 10^3^−10^5^ (BQCV) and 10^2^−10^6^ (DWV) genome copies per gram of flower material. All positive samples came from flowers collected from sites with honeybee apiaries and included the following plant species: (*Asclepias syriaca*, milkweed), (*Monarda spp*., bee balm), (*Trifolium pratense*, red clover), (*Melilotus albus*, white-sweet clover). Of the samples collected in apiaries, 26.7% were positive for DWV, 20% were positive for BQCV and one was positive for both viruses. Honeybee abundance but not bumblebee abundance was greater in sites where we detected viruses on plants (*χ*_1_^2^ = 7.567,P = 0.006; Table 3). Site-level floral density was significantly positively correlated with DWV prevalence (*χ*_1_^2^ = 6.025,P = 0.014) and replicating DWV (*χ*_1_^2^ = 5.461,P = 0.019) in bumble bees. However, floral density was not correlated with BQCV prevalence, BQCV load, replicating BQCV, or DWV load (Table 3).

**Table 3:** Results of the GLMM for virus prevalence on flowering plants showing fixed effects tested. Prevalence is reported as the percentage of flowering plants with viruses detected. Bee abundance was measured as the number of bees (either honeybees or bumblebees) observed per m^2^. Virus species is either deformed wing virus (DWV) or black queen cell virus (BQCV). Floral density was calculated as the number of inflorescences per m^2^. Table shows chi squared value, degrees of freedom (Df) and p-value.

Model/Parameter	*χ*^2^	P
**Virus Prevalence on Flowers**	-	-
*Bombus* Abundance	2.455	0.117
*Apis* Abundance	15.303	<0. 001*
Virus Species	0.2801	0.596
Floral Density	3.315	0.069

Asterisk (*) represents significance.

## Discussion

The higher prevalence of both BQCV and DWV in bumblebees near honeybee apiaries, the lack of finding DWV in bumblebees at sites without honeybees, and the presence of viruses on flowers collected only from sites with apiaries, provide strong support for the hypothesis that RNA viruses are spilling over from managed honeybees into wild bumblebee populations through the use of shared floral resources.

In addition, we detected replicating virus in both *B*. *bimaculatus* and *B*. *vagans*, demonstrating that the bees were carrying active infections and not simply testing positive for virus particles that they contacted but might then be cleared by passing through the gut. Thus, our study confirms viral replication in two bumblebee species, adding to the list of bee species that may be affected by RNA viruses [13,17–20,28,39].

We detected bee viruses on flowers of four different plant species and only found viruses on flowers within honeybee apiaries. These results support the hypothesis that viruses are left behind by foraging honeybees and provide evidence that sites near honeybee apiaries could be hotspots for disease transmission between honeybees and wild bees through the use of shared floral resources.

If transmission of bee viruses occurs through the shared use of flowers, we predicted virus prevalence patterns to be shaped by landscape level floral composition. The prevalence of DWV in bumblebees was higher in sites with high floral density. Sites with a high abundance of floral resources may become hotspots for transmission, as pollinators will converge to forage and bee density and/or comingling may increase. Our results of DWV support this phenomenon whereby the risk of infection was increased for individual foragers in areas of high floral abundance. However, we did not find an effect of floral density on BQCV prevalence. Other factors besides transmission from honeybees at floral resources may be more important for the spread of BQCV in bumblebees. It is likely that BQCV is vertically transmitted, as with honeybees [40], or highly transmissible among nest mates. In captive lab colonies that are positive for BQCV, prevalence within a colony is near 100% (Alger, unpub. data) indicating that rapid dissemination within a colony may occur. This may also explain our observations of high BQCV prevalence as compared to DWV as well as the occurrence of replicating BQCV infections, regardless of apiary presence.

In the bees we sampled, BQCV prevalence and replication was higher for *B*. *bimaculatus* than *B*. *vagans*. Although both species are medium sized, long-tonged bees belonging to the *Pyrobombus* subgenus, *B*. *bimaculatus* queens emerge earlier and establish colonies before *B*. *vagans*. By emerging earlier, *B*. *bimaculatus* may have an increased opportunity of foraging overlap with honeybees and contacting virus particles on flowers. In spring, honeybees intensify their pollen foraging activities to sustain brood rearing. If viruses are transmitted among bees through pollen, early-emerging bumblebees could be at a higher risk for contacting contaminated pollen grains left behind by honeybees. Understanding the temporal variation of virus prevalence among bumblebee species and flowers would help to understand the ecological factors driving virus transmission and infectivity.

Several bumblebee species of Europe, North America, and Asia have suffered dramatic declines. Particularly in North America, pathogens appear to be a chief threat to this group [15]. Overall, we detected DWV in 9.3% of all bumblebees tested which falls between other estimates from Europe where reported prevalence ranged from 3% to 11% [13,17]. However, BQCV prevalence (75.7%) in the bumblebees we tested was 12.5 times higher than UK reports (6%) [13]. Although it is often difficult to directly compare results among studies, we believe this substantial difference is notable given the similarities of sample sizes and sampling efforts between the studies. These differences could be due to bumblebee species susceptibility and/or life history traits that affect exposure to the viruses.

Here, we homogenized and pooled flowers for virus assays. Separately testing petals, nectaries, pollen etc. could help understand where viruses are deposited on flowers and lead to experiments testing how different floral traits influence a plant species’ propensity to harbor and transmit viruses. For example, if viruses are detected in nectaries, antiviral secondary metabolites expressed in the nectar of some plants could reduce virus viability [41]. Further, flowers with deep nectaries could exclude some pollinators and reduce transmission between bee species. Floral morphology that influences bee-flower contact or forager handling time could also affect virus deposition [36]. Future controlled experiments should elucidate how differences in floral traits influence the likelihood for virus deposition and transmission.

Our results showed that both honeybee virus loads and apiary presence are important predictors of virus prevalence in bumblebees. These findings indicate a need for management guidelines that reduce the introduction and spread of bee pathogens. Since viruses can spread in honeybees, even at low virus titers [42], management guidelines should limit apiary activity or increase disease monitoring in critical habitat of sensitive wild bee populations. Although our study focused on two RNA viruses, the spillover of numerous other pests and pathogens from commercial bees is well documented [10,11,14,43,44]. With the increase in global transportation of commercial pollinators, introduced pests and pathogens will continue to pose problems for conservation efforts underlining the need to prevent the introduction of disease through robust monitoring and management practices.

Despite the burgeoning interest in viruses among wild bees, the effects of viruses on non-*Apis* species physiology and fitness are virtually unknown (but see [17,25,45]). RNA viruses may be contributing to the observed declines in bumblebees or bumblebees may serve as tolerant reservoir hosts, facilitating the maintenance of viral infections within the pollinator community at large. In all, knowledge of the effects of RNA viruses and the conditions under which transmission among bee species occurs is critical to a predictive understanding that informs efforts to protect vulnerable species.

## Supporting information

S1 Fig Distribution of site average honeybee DWV load (log transformed). Distribution follows a bimodal distribution with sites either have high (> 10^7^ genome copies) or low (< 10^7^ genome copies) viral loads.
(DOCX)

S2 Fig Box plot of bee abundance across all sites. Bars and points are color coded by bee species: honeybee (*Apis*) or bumblebee (*Bombus*). Bar groupings represents site type: apiary absent or apiary present within 1 km from site. Bee abundance was measured as the number of bees within 5 m of either side of a 100 m transect over a 10-minute period.
(DOCX)

S1 Table Collection site data. Site IDs were assigned for each collection site. Location is provided with latitude and longitude. Sites either had a commercial apiary present (Y) or no apiary nearby (N). Total sampling sizes are given for each of two bumblebee species (*Bombus bimaculatus* and *B*. *vagans*) and honeybees (*Apis mellifera*).
(DOCX)

S2 Table Primers used for the amplification of RNA virus and actin amplicons. (DOCX)

S3 Table Results of the GLMMs showing each model and the fixed effects tested. Table shows chi squared value, degrees of freedom (Df) and p-value. Honeybee abundance was calculated as the number of honeybees observed within 5 m of either side of a 100 m transect over a 10-minute period. Floral density was calculated as the number of inflorescences per m^2^. Bee species was either *Bombus bimaculatus* or *B*. *vagans*. Asterisks represent significance.
(DOCX)

S4 Table Results of the GLMM for DWV prevalence in bumblebees as a function of virus loads in honeybees (high/low), honeybee abundance, and floral density. Prevalence in bumblebees is the percentage of bumblebees with DWV detected. Honeybee virus loads were calculated as the number of virus genome copies per bee and log transformed. Virus loads in honeybees were considered “high” if above 15 (>10^7^ genome copies) and “low” if below 15 (<10^7^ genome copies). Bee abundance was measured as the number of honeybees observed per m^2^. Floral density was calculated as the number of inflorescences per m^2^. Table shows chi squared value, degrees of freedom (Df) and p-value. Asterisks represent significance.
(DOCX)

S1 Data gBlocks gene fragments (integrated DNA technologies) sequence. Virus and actin amplicons are colored for visualizations: Green = DWV, Blue = IAPV, Red = Actin, Yellow = IAPV. Ten random base pairs (uncolored) flank each target of interest.
(DOCX)

S1 Appendix GITC- RNA lysis/stabilization buffer reagents and protocol. (DOCX)
