# Supplementary material for: RNA virus spillover from managed honeybees (Apis mellifera) to wild bumblebees (Bombus spp.)
Source: PLoS One. 2019 Jun 26;14(6):e0217822. doi: 10.1371/journal.pone.0217822 (PMC6594593; doi:10.1371/journal.pone.0217822)
Supplement: S1 Fig — Distribution follows a bimodal distribution with sites either have high (> 107 genome copies) or low (< 107 genome copies) viral loads. (DOCX) [file pone.0217822.s001.docx]

**S1 Fig.** Distribution of site average honeybee DWV load (log transformed).
