# Supplementary material for: RNA virus spillover from managed honeybees (Apis mellifera) to wild bumblebees (Bombus spp.)
Source: PLoS One. 2019 Jun 26;14(6):e0217822. doi: 10.1371/journal.pone.0217822 (PMC6594593; doi:10.1371/journal.pone.0217822)
Supplement: S2 Fig — Bars and points are color coded by bee species: honeybee (Apis) or bumblebee (Bombus). Bar groupings represents site type: apiary absent or apiary present within 1 km from site. Bee abundance was measured as the number of bees within 5 m of either side of a 100 m transect over a 10-minute period. (DOCX) [file pone.0217822.s002.docx]

**S2 Fig. Box plot of bee abundance across all sites.**
