# Supplementary material for: RNA virus spillover from managed honeybees (Apis mellifera) to wild bumblebees (Bombus spp.)
Source: PLoS One. 2019 Jun 26;14(6):e0217822. doi: 10.1371/journal.pone.0217822 (PMC6594593; doi:10.1371/journal.pone.0217822)
Supplement: S1 Table — Site IDs were assigned for each collection site. Location is provided with latitude and longitude. Sites either had a commercial apiary present (Y) or no apiary nearby (N). Total sampling sizes are given for each of two bumblebee species (Bombus bimaculatus and B. vagans) and honeybees (Apis mellifera). (DOCX) [file pone.0217822.s003.docx]

S1 Table. Collection site data.

| **site ID** | **latitude** | | **longitude** | | **Apiary present?** | | ***Bombus bimaculatus*** | ***Bombus vagans*** | ***Apis mellifera*** |
| --- | --- | --- | --- | --- | --- | --- | --- | --- | --- |
| JOSH | | 44.859642 | | -72.408081 | | Y | 0 | 13 | 10 |
| FERL | | 44.948248 | | -73.082924 | | Y | 10 | 10 | 10 |
| ROCK | | 44.849911 | | -72.942441 | | N | 11 | 8 | 10 |
| MART | | 44.736855 | | -73.086848 | | Y | 10 | 13 | 10 |
| ONE | | 44.336968 | | -73.150093 | | Y | 10 | 8 | 10 |
| BOST | | 44.369755 | | -73.242064 | | Y | 11 | 10 | 10 |
| PAT | | 44.158423 | | -73.339091 | | Y | 10 | 0 | 10 |
| SAND | | 44.654202 | | -73.16209 | | N | 10 | 10 | 5 |
| FLAN | | 44.237572 | | -73.231302 | | N | 10 | 0 | 0 |
| SWAN | | 44.931132 | | -73.091239 | | N | 10 | 10 | 10 |
| WHAL | | 44.326216 | | -73.278147 | | Y | 0 | 11 | 10 |
| COL | | 44.550141 | | -73.12475 | | N | 10 | 11 | 10 |
| CLERK | | 44.807917 | | -72.447151 | | N | 11 | 10 | 0 |
| MUGE | | 44.672081 | | -72.599161 | | N | 10 | 8 | 10 |
| CIND | | 44.50658 | | -72.626181 | | N | 10 | 0 | 9 |
| HOGB | | 44.682381 | | -72.773484 | | N | 10 | 10 | 10 |
| NEK | | 44.950872 | | -71.830196 | | N | 0 | 13 | 0 |
| TIRE | | 44.87368 | | -72.051344 | | N | 8 | 12 | 0 |
| RICE | | 44.925435 | | -72.969001 | | N | 10 | 15 | 9 |
