# Supplementary material for: RNA virus spillover from managed honeybees (Apis mellifera) to wild bumblebees (Bombus spp.)
Source: PLoS One. 2019 Jun 26;14(6):e0217822. doi: 10.1371/journal.pone.0217822 (PMC6594593; doi:10.1371/journal.pone.0217822)
Supplement: S2 Table — (DOCX) [file pone.0217822.s004.docx]

**S2 Table.** Primers used for the amplification of RNA virus and actin amplicons.

| Primer | 5’ to 3’ Sequence | Product Size (bp) | Annealing Temp (^o^C) | Reference |
| --- | --- | --- | --- | --- |
| DWV-F | TTCATTAAAGCCACCTGGAACATC | 136 | 53 | [1] |
| DWV-R | TTTCCTCATTAACTGTGTCGTTGA |  |  |  |
| BQCV-F | TTTAGAGCGAATTCGGAAACA | 140 | 51 | [1] |
| BQCV-R | GGCGTACCGATAAAGATGGA |  |  |  |
| IAPV-F | CCATGCCTGGCGATTCAC | 203 | 47 | [1] |
| IAPV-R | CTGAATAATACTGTGCGTATC |  |  |  |
| Actin-F | CGTGCCGATAGTATTCTTGC | 138 | 56 |  |
| Actin-R | CCATTGTCAACTACGAGTGC |  |  |  |

References

1. Traynor KS, Rennich K, Forsgren E, Rose R, Pettis J, Kunkel G, et al. Multiyear survey targeting disease incidence in US honey bees. Apidologie. 2016;47: 325–347. doi:10.1007/s13592-016-0431-0
