# Supplementary material for: RNA virus spillover from managed honeybees (Apis mellifera) to wild bumblebees (Bombus spp.)
Source: PLoS One. 2019 Jun 26;14(6):e0217822. doi: 10.1371/journal.pone.0217822 (PMC6594593; doi:10.1371/journal.pone.0217822)
Supplement: S3 Table — Table shows chi squared value, degrees of freedom (Df) and p-value. Honeybee abundance was calculated as the number of honeybees observed within 5 m of either side of a 100 m transect over a 10-minute period. Floral density was calculated as the number of inflorescences per m2. Bee species was either Bombus bimaculatus or B. vagans. Asterisks represent significance. (DOCX) [file pone.0217822.s005.docx]

| Model/Parameter | $\boldsymbol{\chi}^{\boldsymbol{2}}$ | Df | P |
| --- | --- | --- | --- |
| BQCV Prevalence | - | - | - |
| *Apis* Abundance | 3.868 | 1 | **0.049*** |
| Floral Density | 0.847 | 1 | 0.357 |
| *Bombus* Species | 16.783 | 1 | **<0.001*** |
| DWV Prevalence | - | - | - |
| *Apis* Abundance | 5.856 | 1 | **0.016*** |
| Floral Density | 6.303 | 1 | **0.012*** |
| *Bombus* Species | 0.045 | 1 | 0.831 |
| BQCV Load | - | - | - |
| *Apis* Abundance | 1.887 | 1 | 0.169 |
| Floral Density | 2.159 | 1 | 0.142 |
| *Bombus* Species | 16.935 | 1 | **<0.001*** |
| DWV Load | - | - | - |
| *Apis* Abundance | 1.490 | 1 | 0.222 |
| Floral Density | 0.218 | 1 | 0.640 |
| *Bombus* Species | 0.0003 | 1 | 0.987 |
| BQCV Negative Strand | - | - | - |
| *Apis* Abundance | 0.539 | 1 | 0.463 |
| Floral Density | 0.119 | 1 | 0.730 |
| *Bombus* Species | 14.447 | 1 | **<0.001*** |
| DWV Negative Strand | - | - | - |
| *Apis* Abundance | 7.037 | 1 | **0.008*** |
| Floral Density | 6.812 | 1 | **0.009*** |
| *Bombus* Species | 0.334 | 1 | 0.564 |

**S3 Table.** Results of the GLMMs showing each model and the fixed effects tested.
