# Supplementary material for: RNA virus spillover from managed honeybees (Apis mellifera) to wild bumblebees (Bombus spp.)
Source: PLoS One. 2019 Jun 26;14(6):e0217822. doi: 10.1371/journal.pone.0217822 (PMC6594593; doi:10.1371/journal.pone.0217822)
Supplement: S4 Table — Prevalence in bumblebees is the percentage of bumblebees with DWV detected. Honeybee virus loads were calculated as the number of virus genome copies per bee and log transformed. Virus loads in honeybees were considered “high” if above 15 (>107 genome copies) and “low” if below 15 (<107 genome copies). Bee abundance was measured as the number of honeybees observed per m2. Floral density was calculated as the number of inflorescences per m2. Table shows chi squared value, degrees of freedom (Df) and p-value. Asterisks represent significance. (DOCX) [file pone.0217822.s006.docx]

**S4 Table.** Results of the GLMM for DWV prevalence in bumblebees as a function of virus loads in honeybees (high/low), honeybee abundance, and floral density.

| Model/Parameter | $\boldsymbol{\chi}^{\boldsymbol{2}}$ | Df | P |
| --- | --- | --- | --- |
| DWV Prevalence by *Apis* | - | - | - |
| *Apis* Abundance | 3.786 | 1 | 0.052 |
| *Apis* DWV load | 8.068 | 2 | **0.018*** |
| Floral Density | 3.323 | 1 | 0.068 |
