# Supplementary material for: RNA virus spillover from managed honeybees (Apis mellifera) to wild bumblebees (Bombus spp.)
Source: PLoS One. 2019 Jun 26;14(6):e0217822. doi: 10.1371/journal.pone.0217822 (PMC6594593; doi:10.1371/journal.pone.0217822)
Supplement: S1 Data — Virus and actin amplicons are colored for visualizations: Green = DWV, Blue = IAPV, Red = Actin, Yellow = IAPV. Ten random base pairs (uncolored) flank each target of interest. (DOCX) [file pone.0217822.s007.docx]

**S1 Data.** gBlocks Gene Fragments (Integrated DNA Technologies) sequence.

GGACGGACAGTTCATTAAAGCCACCTGGAACATCAGGTAAGCGATGGTTGTTTGACATTGAGCTACAAGACTCGGGATGTTATCTCTTGCGTGGAATGCGTCCCGAACTTGAGATTCAATTATCAACGACACAGTTAATGAGGAAAACCATGTACGCCATGCCTGGCGATTCACAACAAGAAAGCAATACTCCCAATGTACACAACACGGAACTCGCTTCGTCAACTAGTGAAAACTCGGTTGAGACCCAAGAAATCACAACCTTTCATGATGTGGAAACTCCAAATAGGATCGATACCCCCATGGCTCAGGATACTTCATCGGCTAGGAACATGGATGATACGCACAGTATTATTCAGCTTCCCTGCTCGTGCCGATAGTATTCTTGCGGTGTCTCTTTGCCGATCAACGATCGTGTACTTTGTTGGTTACCTTCGATTCTAAAAGATAACTCAATAAACCAAACATGTGTGACGAAGAAGTTGCTGCACTCGTAGTTGACAATGGCGTCCACCTGTTTAGAGCGAATTCGGAAACATTTTACTATAGTTCAGGTCGGAATAATCTCGATATAGCCACTTCACCTCCTTCCATCAATCGCTACTATGCGGTAGGTGCGGGAGATGATATGGACTTTTCCATCTTTATCGGTACGCC ATGAGCGCCA
