# Supplementary material for: RNA virus spillover from managed honeybees (Apis mellifera) to wild bumblebees (Bombus spp.)
Source: PLoS One. 2019 Jun 26;14(6):e0217822. doi: 10.1371/journal.pone.0217822 (PMC6594593; doi:10.1371/journal.pone.0217822)
Supplement: S1 Appendix — (DOCX) [file pone.0217822.s008.docx]

**S1 Appendix.** GITC-RNA lysis/stabilization buffer and protocol.

**Reagents** **Amount added to 1 L beaker**

1M Tris , pH 7.0 (50mM) 50 mL

Ultra Pure 0.5M EDTA, pH 8.0 (20mM) 40 mL

Triton –x100 (1.3%) 13 ul

Guanidine thiocyanate (5.25M) 620.34 g

Place a large magnetic stir bar into a 1L beaker.

Add reagents as above.

Bring up to 1000 mL with **nuclease-free water.**

Stir until completely dissolved.

Aliquot into RNAse free falcon tubes.
